# Opportunistic proteolytic processing of carbonic anhydrase 1 from Chlamydomonas in Arabidopsis reveals a novel route for protein maturation

**DOI:** 10.1093/jxb/erw044

**Published:** 2016-02-24

**Authors:** Parijat S. Juvale, Ryan L. Wagner, Martin H. Spalding

**Affiliations:** ^1^Department of Plant Pathology and Microbiology, Iowa State University, Ames, IA 50011, USA; ^2^Department of Biology, Millersville University, Millersville, PA 17551, USA; ^3^Department of Genetics, Development and Cell Biology, Iowa State University, 202 Catt Hall, Ames, IA 50011–1301, USA

**Keywords:** Carbonic anhydrase, *Chlamydomonas*, *reinhardtii*, endomembrane system, post-translational modifications, pre-protein processing, proteases.

## Abstract

Proteolytic processing of secretory proteins to yield an active form generally involves specific proteolytic cleavage of a pre-protein. Multiple specific proteases have been identified that target specific pre-protein processing sites in animals. However, characterization of site-specific proteolysis of plant pre-proteins is still evolving. In this study, we characterized proteolytic processing of Chlamydomonas periplasmic carbonic anhydrase 1 (CAH1) in Arabidopsis. CAH1 pre-protein undergoes extensive post-translational modification in the endomembrane system, including glycosylation, disulfide bond formation and proteolytic removal of a peptide ‘spacer’ region, resulting in a mature, heterotetrameric enzyme with two large and two small subunits. We generated a series of small-scale and large-scale modifications to the spacer and flanking regions to identify potential protease target motifs. Surprisingly, we found that the endoproteolytic removal of the spacer from the CAH1 pre-protein proceeded via an opportunistic process apparently followed by further maturation via amino and carboxy peptidases. We also discovered that the spacer itself is not required for processing, which appears to be dependent only on the number of amino acids separating two key disulfide-bond-forming cysteines. Our data suggest a novel, opportunistic route for pre-protein processing of CAH1.

## Highlight

Protein maturation in plants can proceed via a pathway involving opportunistic endoprotease targeting of an unfolded or poorly folded polypeptide region of a pre-protein.

## Introduction

Site-specific limited proteolysis is a selective mechanism that can be either co-translational or act in concert with other post-translational modifications in many cellular processes such as embryogenesis (Zhu *et al.*, 2002), stress response (Thornberry and Lazebnik, 1998; Liu *et al.*, 2007; Polge *et al.*, 2009), maturation of inactive hormones, neuropeptides and growth factors (Rholam and Fahy, 2009; Seidah, 2011), maturation of small secreted plant signalling peptides (Matsubayashi, 2011), and intracellular protein targeting (Martoglio and Dobberstein, 1998). Typically, genes are first transcribed in the nucleus to generate mRNA that is in turn translated in the cytosol to form a pre-protein. The endomembrane targeting signal peptide, which is present at the N-terminus of many pre-proteins, is identified by a signal recognition particle (SRP), targeting the nascent polypeptide to the endomembrane, where signal peptidase cleaves the signal peptide as the polypeptide chain gains entry into the endoplasmic reticulum (ER). During the journey through the secretory pathway that includes the ER, Golgi bodies and secretory vesicles, secretory proteins undergo a series of post-translational modifications required to generate correctly folded, functional proteins. In the current research, we are studying post-translational modification, especially the proteolysis, of a secreted protein, periplasmic carbonic anhydrase 1 (CAH1) from a unicellular eukaryotic green alga, *Chlamydomonas reinhardtii* (Chlamydomonas, hereafter).

**Fig. 1.**
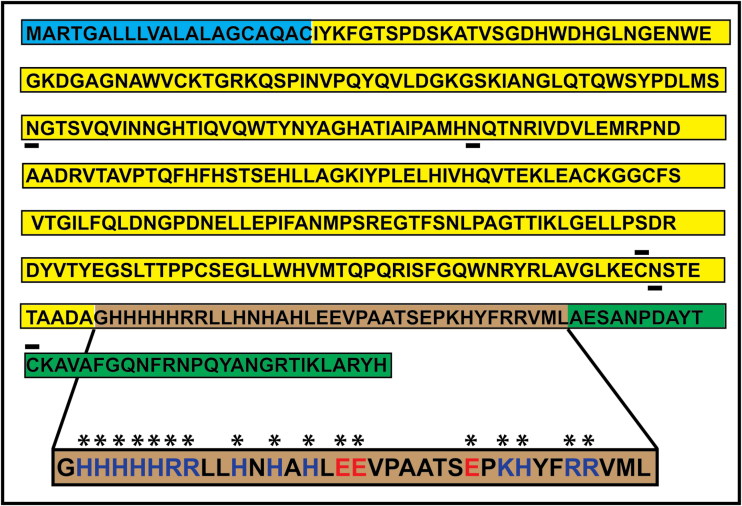
Amino acid sequence of the CAH1 pre-protein. The endoplasmic reticulum targeting peptide sequence is shown in blue, the large subunit in yellow, and the small subunit in green. The spacer region is shown in brown, with charged amino acids indicated by blue (cationic) and red (anionic) letters and emphasized by asterisks. The disulfide bond-forming cysteine residues are shown with a bar above, while predicted glycosylated asparagine residues are indicated with a bar below.

Carbonic anhydrase is a universal enzyme that catalyses reversible hydration of CO_2_ (CO_2_+H_2_O↔HCO_3_^−^+H^+^). Chlamydomonas acclimates to limiting CO_2_ concentration via an acclimation response known as the CO_2_-concentrating mechanism (CCM). One component of this CCM is induction of a nuclear gene, *CAH1*, producing the CAH1 protein (Fukuzawa *et al.*, 1990, Fujiwara *et al.*, 1990). The primary product of *CAH1* is a 41.6kDa pre-protein, which undergoes intense post-translational modification to give rise to a glycosylated heterotetramer assembled with disulfide bridges, which ultimately is secreted (Fukuzawa *et al.*, 1990). CAH1 (Fig. 1) has a 20-amino-acid signal peptide that targets the pre-protein to the ER and is removed presumably during its insertion into the ER. During further post-translational processing, a 35-amino-acid peptide stretch referred to as the ‘spacer’ is proteolytically removed to yield one large and one small subunit. Oligomerization results in the formation of a heterotetramer comprising two large subunits and two small subunits held together by disulfide bridges (Kamo *et al.*, 1990). There are three asparagine-linked glycosylation sites, Asn–X– Thr/Ser (Fig. 1), in the large subunit, which are all glycosylated (Ishida *et al.* 1993). Various aspects of this unique post translational processing are yet to be fully understood. For example, it is still not known whether any specific amino acid sequence is required for recognition and cleavage of the pre-protein by an endopeptidase in the secretory pathway or in which compartment of the cell this cleavage occurs.

In most known cases of limited and specific proteolysis, proteases recognize specific amino acid sequences in the polypeptides. For example, kex2p protease, which is responsible for maturation of the α-mating factor in yeast, recognizes and cuts at the C-terminus of a dibasic site, usually -RR- or -KR- (Kjeldsen, 2000). Similarly, the subtilases (SBTs) or pro-protein convertases (PCs) in mammals also rely on the occurrence of dibasic sites in their target polypeptides (Rockwell and Thorner, 2004; Rholam and Fahy, 2009; Seidah, 2011). Site specific proteolysis has been reported in plants as well. Study of a virally encoded antifungal toxin (KP6) in transgenic tobacco suggests the existence of KEX2-like protease in the secretory pathway (Kinal *et al.*, 1995, Jiang and Rogers, 1999). Maturation of the peptide hormone Arabidopsis rapid alkalinazation factor 1 (AtRALF1) and the inactivation of the tomato wound signalling peptide systemin also indicate the presence of proteolytic enzymes in plants that target dibasic sites (Narvaez-Vasquez and Ryan, 2004; Matos *et al.* 2008). Also, research on antimicrobial peptides derived from *Impatiens balsamina* seeds predicts, but does not demonstrate, the involvement of an endoprotease targeting a diacidic (-EE-/-ED-) amino acid motif as well as the involvement of aminopeptidases and carboxypeptidases in the processing of a single pre-protein to multiple functional peptides (Tailor *et al.*, 1997). Further research involving chimeric polyproteins connected by a ‘linker peptide’ originating from the same pre-protein from *I. balsamnia* demonstrated the existence of proteases required for processing of the *I. balsamnia* polyproteins in the secretory pathway of *Arabidopsis thaliana* (hereafter, Arabidopsis) (Francois *et al.*, 2002). Successful expression of processed CAH1 in tobacco and maize also indicates the presence of protease activity in the secretory pathway of plants (Roberts and Spalding, 1995). Multiple research articles have been published in recent years that suggest the presence of proteolytic enzymes targeting specific amino acid domains within pro-proteins in various plant species (Matsubayashi, 2011; Schaller *et al.*, 2012), although other specific amino acid motifs may also serve as protease processing sites.

We envisioned using the CAH1 pre-protein processing system for coordinated expression of two or more proteins in Arabidopsis by inserting the CAH1 spacer between their coding sequences. However, before proceeding, we reasoned it was essential to identify the determining factors for the proteolytic removal of the spacer. Therefore, in the research described here, we developed various strategies and constructs to explore the post-translational processing of CAH1 in Arabidopsis. We hypothesized that proteolysis of the CAH1 pre-protein is controlled by specific conserved motifs targeted by specific proteases. Our goal in the research described was to identify these specific targeted motifs within the spacer peptide. To this end, we designed a wide variety of constructs with specific amino acid sequence and larger scale sequence modifications to the spacer and the flanking regions of the CAH1 large and small subunits. To our surprise, our hypothesis was proved incorrect, and it appears that the proteolytic processing of CAH1 involves an opportunistic endoproteolytic cleavage followed by further proteolytic digestion by carboxy- and aminopeptidases.

## Materials and methods

### Development of constructs

The CAH1 lacking endomembrane targeting sequence was amplified using the forward primer P7 (5′ GAATTCGCCATGGCTTGC AT 3′) and the reverse primer P11 (5′ GAGCTCTAGACTTTAGTGAT 3′) using previously cloned CAH1 full length cDNA from *Chlamydomonas reinhardtii* (Roberts and Spalding, 1995) In a separate experiment, previously cloned endomembrane targeting peptide coding sequence from Arabidopsis ‘basic chitinase’ gene (AT3G12500) fused with green fluorescent protein (GFP) (Y Nakamura and MH Spalding, unpublished results) was ligated with the BSSK4 vector between *Bam*H1 and *Sst*1 restriction sites. This construct had *Eco*R1 and *Nco*1 restriction sites between the endomembrane targeting sequence and the GFP. Finally, GFP was replaced with CAH1 in the BSSK4 vector giving the final GrCon1 construct in which CAH1 was fused with an Arabidopsis specific endomembrane targeting peptide coding sequence with the *Bam*H1 site at the 5′ end, and *Xba*1 and *Sst*1 sites at the 3′ end, while *Eco*R1 and *Nco*1 sites separated CAH1 coding sequence from the endomembrane targeting sequence (Supplementary Fig. S1 at *JXB* online). The rest of the constructs were developed using standard molecular biology methods using the Gr1Con1 construct as a template.

The method used to name all the developed constructs is as follows. The constructs were grouped based on the location and nature of their modifications. Group 1 constructs (Gr1…) have modification at the junctions between the large and/or small subunit, and the spacer peptide. Group 2 constructs (Gr2…) have modifications in the predicted target sites within the spacer peptide. Group 3 constructs (Gr3…) have large-scale deletions within the spacer. Group 4 constructs (Gr4…) have large-scale modifications within the spacer peptide. Group 5 constructs (Gr5…) have deletions in the large and/or small subunits immediately flanking the spacer peptide. The last group of constructs, Group 6 (Gr6…), lack the spacer peptide entirely and also have progressively reduced numbers of amino acids separating the disulfide bond-forming cysteine residues of the large and small subunits. Constructs within each group are numbered in increasing order.

The strategies followed to develop the constructs are discussed in detail in Supplementary materials and methods along with the primer sequences. In short, all the constructs were developed first in BSSK4 vector that contains ampicillin resistance for selection of transformed *E. coli*, and were later moved to the binary vector, pCB302-3 (Xiang *et al.*, 1999), which contains a kanamycin resistance cassette for selection of transformed *E. coli* and a Bar gene for selection of transgenic plants. This binary vector has a multi-cloning site flanked by a 35S promoter and a Nos terminator for constitutive expression of the construct in plants, resulting in constitutive expression driven by the 35S promoter. In each case, successful ligation of the construct with the binary vector was confirmed by sequencing (using the primer P3: 5′ ACTCCACCTCGGAGCACCTG 3′) part of CAH1 PCR amplified with the forward primer P7 and reverse primer P11.

After the sequence confirmation, the modified pCB302-3 vector was transferred by electroporation into *Agrobacterium* strain C58 using gentamycin, rifampicin and kanamycin resistance genes as selection markers. The transformation was confirmed by amplifying a part of the CAH1 construct with the forward primer, P7, and the reverse primer, P11. The transformed *Agrobacterium* cells were used for the generation of the transgenic plants.

### Transgenic plant development

*Arabidopsis thaliana*, ecotype Columbia, wild-type plants were grown in continuous day conditions in the greenhouse at 22 ºC. After transformation by the standard *Agrobacterium*-mediated floral dip method (Clough and Bent, 1998), the wild-type plants were allowed to develop seeds for about 20 d, then subjected to dehydration and seed collection. T_0_ seeds were sown on fresh soil, and transgenic plants were selected at the seedling stage by spraying with a herbicide, Liberty. Following isolation of genomic DNA from the transgenic plants using a standard CTAB extraction method (Murray and Thompson, 1980), the presence of the construct in each case was confirmed by PCR amplification using the primers P7 and P11, purification (QIAquick PCR purification kit; Qiagen) and sequencing (ISU sequencing facility) using forward primer, P3, of the PCR product.

### Confirmation of protein expression

The protein expression by individual transgenic plants was confirmed using a standard western immunoblot protocol. Total protein was extracted using protein extraction buffer (50mM Tris–HCl 7.5, 50mM KCl, 4mM EDTA, 1 µg ml^–1^ PMSF) and the protein concentration determined using the Bradford assay (Noble and Bailey, 2009). Protein (a uniform amount for all lanes of each gel) was heat denatured and treated with 2-mercaptoethanol to reduce/cleave disulfide bonds, subjected to SDS polyacrylamide gel electrophoresis (SDS-PAGE) on 12.5% polyacrylamide gels (Guide to Protein Purification), and electrophoretically transferred to PVDF membranes using semidry transfer (Bio-Rad). The membranes were probed with rabbit anti-CAH1 polyclonal antibody (Roberts and Spalding, 1995), followed by a goat anti-rabbit IgG secondary antibody conjugated with horseradish peroxidase, and the cross-reactive bands were visualized by chemiluminescence (Yakunin and Hallenbeck, 1998). To verify formation of heterotetrameric conformation, in some cases protein was not treated with 2-mercaptoethanol. The molecular masses of the SDS-PAGE bands representing large subunits of various constructs were estimated by calculating relative migration distance (*R*_f_) of the particular protein band using a standard curve generated from molecular mass standards (Weber and Osborn, 1975).

### Enzyme activity

Carbonic anhydrase activity was assayed by measuring the time interval required for a pH change from 8.5 to 7.5 in 3ml of 25mM barbital (pH 9.5 with KOH), 2ml CO_2_-saturated water and 100 µg of protein (determined by the Bradford assay) extracted from individual plants in protein extraction buffer (50mM Tris–HCl 7.5, 50mM KCl, 4mM EDTA, 1 µg ml^–1^ PMSF). The time required for the pH change was measured three times for each individual sample and three times for the buffer alone and the average calculated for each. Enzyme activity in CA units/100 µg of protein was calculated as: [(average time for the buffer/average time for the individual sample)–1]×10. The formula was applied for each of the three readings for individual samples and the mean activity was calculated.

### Protein purification, sequence and peptide analyses

CAH1 was purified from Arabidopsis plants by affinity chromatography on *p*-aminomethylbezenesulfonamide-agarose as previously described (Roberts and Spalding, 1995). The CAH1 content of collected fractions was determined by western immunoblot analysis.

Protein bands of interest were excised from gels. The gel slices were cut into pieces and washed with 500 μl of 100mM NH_4_HCO_3_ for 1h with agitation. The gel pieces were further washed three times with 20mM NH_4_HCO_3_–50% (v/v) acetonitrile. After dehydration in acetonitrile, the gel pieces were incubated with 10mM dithiothreitol in 100mM NH_4_HCO_3_ for 30min at 60 ºC, followed by reaction with 55mM iodoacetamide in 100mM NH_4_HCO_3_ for 30min in the dark at room temperature for reducing and alkylation. The gel pieces were washed with 20mM NH_4_HCO_3_ and dehydrated with acetonitrile, and the washing and dehydration procedure was repeated once. After drying at 37 ºC for 30min, the gel pieces were swollen in a digestion buffer containing 20mM NH_4_HCO_3_ and 10ng μl^–1^ of tosyl phenylalanyl chloromethyl ketone (TPCK)-treated sequence-grade trypsin (Promega, Madison, WI, USA) at 4 ºC. After 30 minutes, the supernatant was removed and replaced with 20 μl of 20mM NH_4_HCO_3_ to keep them wet during overnight digestion at 37 ºC. The supernatant was collected, and the gel pieces were further extracted by two changes of 1% acetic acid in 50% acetonitrile. The solutions were combined with the supernatant and dried. One microlitre of matrix solution (α-cyano-4-hydroxycinnamic acid (CHCA) 10mg/ml in 50% acetonitrile (CH_3_CN)/0.1% trifluoroacetic acid (TFA)) was used to dissolve the peptides and applied on a matrix-assisted laser desorption/ionization (MALDI) target plate.

MALDI–time of flight mass spectrometry (TOF MS)/MS analyses were performed by the Iowa State University Proteomics Facility using a QSTAR XL quadrupole TOF mass spectrometer (AB/MDS Sciex, Toronto, Canada) equipped with an oMALDI ion source and operated in the positive ion mode. Mass spectra for MS analysis were acquired over *m*/*z* 500–4 000, and MS/MS acquisition was performed against most intensive ions after every regular MS acquisition. The molecular ions were selected by information-dependent acquisition in the quadrupole analyser and fragmented in the collision cell.

Column purified CAH1 protein was separated by denaturing SDS-PAGE, transferred to immobilon transfer membrane (0.2 µm pore size, Millipore), and stained with Coomassie Brilliant Blue R-250. The stained band corresponding to the predicted molecular mass of the small subunit was subjected to N-terminal sequencing on a Procise protein sequencer (model 494, Applied Biosystems, Foster City, CA, USA) by the ISU Protein Facility.

In order to evaluate whether any unprocessed small subunit was still connected to the large subunit, affinity purified CAH1 protein was separated by denaturing SDS-PAGE gel and stained with Coomassie Brilliant Blue R-250. The stained band corresponding to the large subunit was submitted to the ISU Protein Facility, where it was subjected to trypsin digestion, followed by separation of tryptic peptides by reverse phase HPLC (1mm by 250mm, C18 column from Vydac; Beckman model 126AA). MALDI-TOF analysis was performed on the resolved peptide fractions (Finnaga Met Dynax). The peptides from fractions that matched predicted molecular mass of peptides from the small subunit were subjected to N-terminus sequence analysis.

## Results

### Arabidopsis can express active CAH1

**Fig 2.**
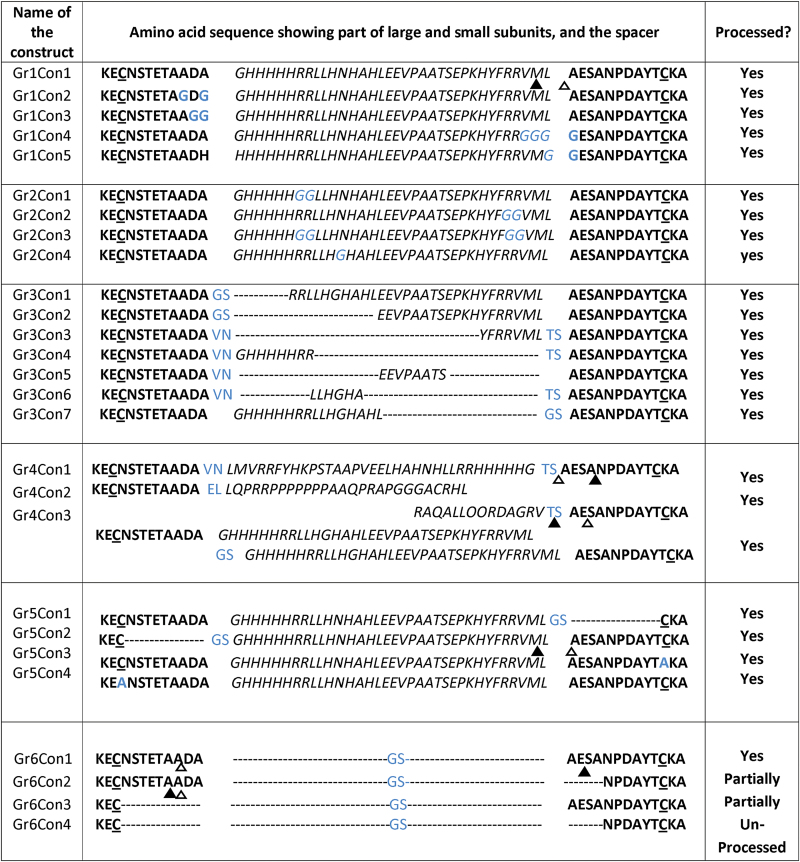
Amino acid sequence of the various modified CAH1 constructs. The spacer sequence is italicized, and the flanking sequences of the small and large subunits are in bold. Substitutions and additions are in blue font, deletions are indicated by dashes, and the key disulfide-bond forming cysteines are underlined. Determined cleavage sites of the small subunit are indicated by triangles, solid for the major peptide and hollow for minor peptides. Status of proteolytic cleavage of CAH protein for each construct is indicated in the last column. Estimated molecular mass of the upper band of Gr6Con1 was 37 kDA and of the lower band was 35kDa. Estimated molecular masses of the bands observed for partially processed Gr6Con2, in decreasing size, are 42, 40, 37, and 34kDa. Estimated molecular masses for partially processed Gr6Con3, in decreasing size, are 37 and 34kDa, while that of unprocessed Gr6Con4 was 37kDa.

**Fig. 3.**
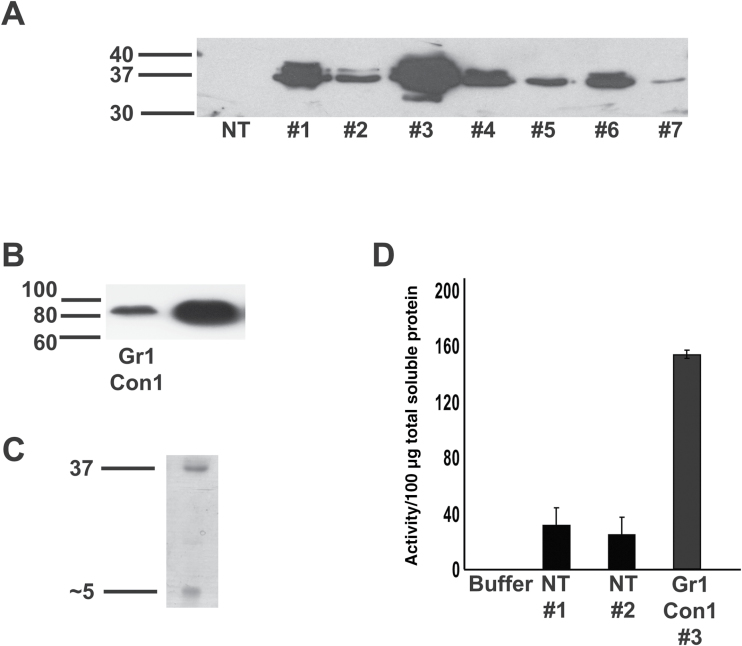
Arabidopsis expresses processed and active CAH1. (A) Western immunoblot of seven individual T1 transgenic Arabidopsis plants expressing Gr1Con1 and an untransformed control (NT). All transgenic lines show apparent double bands for CAH1, apparently due to differential glycosylation. Each lane contains 30 µg total soluble protein. (B) Nonreducing, western immunoblot analysis of total soluble protein from a transgenic Arabidopsis plant expressing Gr1Con1 with intact disulfide bonds. Ten micrograms of total soluble protein in each lane. (C) Coomassie stained PVDF membrane showing the large subunit at molecular mass 37kDa and the small subunit at 5kDa, from affinity purified Gr1Con1 construct. (D) Carbonic anhydrase activity for transgenic plant no. 3 expressing Gr1Con1 and two untransformed plants, NT-1 and NT-2. Total soluble protein used for the activity assay was 100 µg.

Successful expression of CAH1 protein from wild-type construct Gr1Con1 (Fig. 2) was confirmed by western blot, which often showed two similar-sized bands running very close together, with the lower band more intense (Fig. 3A and Supplementary Fig. S2). Earlier studies reported a double band for the large subunit of *Chlamydomonas reinhardtii* CAH1 on SDS-PAGE with a molecular mass difference of approximately 1.5kDa (Kamo *et al.*, 1990). Subsequent analysis demonstrated that this difference was not due to alternative processing of the large subunit, but rather to differential glycosylation of the three large subunit asparagine residues (Ishida, *et al.*, 1993). We also observed the same pattern for this protein expressed in tobacco (Roberts and Spalding, 1995). Thus, the double bands we observed on our western immunoblots most likely result from differential glycosylation of the large subunit. The apparent molecular mass of the lower, more intense band was estimated to be 37kDa, matching the previously reported size (Ishida *et al.*, 1993). The CAH1 expressed in Arabidopsis also assembled into a form consistent with its native heterotetrameric form, as demonstrated with non-reducing SDS-PAGE (Fig. 3B). The small subunit was not detectable in western immunoblots, possibly due to the inability of the primary antibody to interact with the small subunit. The presence of the small subunit was confirmed by SDS-PAGE of affinity purified CAH1. The molecular mass of the small subunit was observed to be approximately 5–8kDa (Fig. 3C), and N-terminal sequence analysis found heterogeneity in the N-terminal sequence, beginning with LAESAN most commonly but in a minority of the cases beginning with AESANP (Fig. 2 and Table 1). These results confirm that the small subunit is proteolytically cleaved from the pre-protein. N-terminal sequencing of the large subunit (Table 1) also confirmed that the N-terminal signal peptide was removed from the large subunit as expected. A far higher carbonic anhydrase enzyme activity was observed in the transgenic plants relative to the control, non-transformed plants, confirming the expressed CAH1 was active (Fig. 3D).

**Table 1.** Major and minor N-terminal sequences of the CAH1 large subunit (first three columns) and of the CAH1 small subunit (second three columns) The construct names, major peptide sequence and minor peptide sequence, respectively, are indicated in the three columns of each set. The parentheses indicate expected amino acids which could not be confirmed due to ambiguous signals.

**CAH1 large subunit N-terminal sequences**			**CAH1 small subunit N-terminal sequences**		
**Construct name**	**Major peptide**	**Minor peptide**	**Construct name**	**Major peptide**	**Minor peptide**
Gr1Con1	(C)IYKFGT	EFAMA(C)I	Gr1Con1	LAESAN	AESANP
Gr5Con2	(C)IYKFGT	(E)FAMA(C)	Gr4Con1	NPDAYT	AESANPD
Gr6Con1	(C)IYKFGT	EFAMA(C)I	Gr4Con2	SAESAN	SANPDAYT
Gr6Con2 Upper two bands	(C)IYKFG	(E)FAMA(C)	Gr5Con2	L(A)ESANPD	AESANPD
Gr6Con2 Lower two bands	(C)IYKFGT	EFAMA(C)I	Gr6Con1	SANPDAY	DAGSAES
Gr6Con3 Upper band	(C)IYKFG	(E)FAMA(C)	Gr6Con2	ADAGSNP	(D)(A)GSNPD
Gr6Con3 Lower band	(C)IYKF(G)	(E)FAMA(C)	Gr6Con3	Not detected	
Gr6Con4	(C)IYKFGT	EFAMA(C)I	Gr6Con4	Not detected	

### The specific peptide sequence separating the large and small subunits of CAH1 may not be critical

In order to identify any conserved motifs targeted by proteases cleaving the small subunit from the CAH1 pre-protein, we designed a wide variety of constructs with specific modifications to the predicted amino acid sequence (Fig. 2). In the first group of modifications (Group 1), we disrupted the amino acid sequence across the junction sites between the spacer and the large and small subunits by deleting, adding or replacing amino acid residues. In the second group (Group 2), we specifically modified sequence motifs within the spacer representing potential, characterized proteolytic recognition sequence motifs. Because of the obvious dibasic sites evident in the spacer, these constructs were designed to disrupt dibasic (-RR-) sites and the lone asparagine residue to test for any essential role played by them in proteolytic cleavage of CAH1. In the third group (Group 3), we expressed CAH1 constructs containing a series of truncated spacer peptides with sequential deletions designed to reveal specific proteolytic recognition sequences within the spacer necessary for proteolytic cleavage. Somewhat surprisingly, none of the Group 1, 2 or 3 constructs disrupted the proteolytic processing of CAH1 pre-protein (Supplementary Fig. S3A–C).

**Fig. 4.**
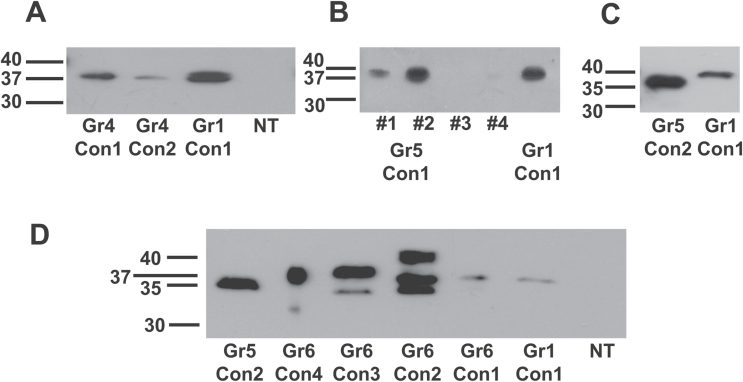
The length rather than the sequence of the peptide separating disulfide bond forming cysteines controls proteolytic cleavage of the small subunit. (A) Western immunoblot analysis of transgenic Arabidopsis plants expressing Gr4Con1 and Gr4Con2, along with Gr1Con1 and untransformed Arabidopsis (NT). (B) Western immunoblot analysis of transgenic Arabidopsis plants expressing Gr5Con1 construct. (C) Western immunoblot analysis of transgenic Arabidopsis plant expressing Gr5Con2 construct. (D) Western immunoblot analysis for size comparison between the large subunits of the group 6 constructs and Gr5Con2. For (A), (B) and (C) each lane contains 10 µg total soluble protein. Amount of proteins added in each lane in (D) are as follows: Gr5Con2, 28 µg; Gr6Con4, 5 µg; Gr6Con3, 40 µg; Gr6Con2, 36 µg; Gr6Con1, 6 µg; Gr1Con1, 16 µg; non-transformed plant, 18 µg.

Specific motifs targeted by proteases could, in theory include any amino acid sequence, so to further probe the nature of CAH1 pre-protein processing, a group of constructs (Group 4; Fig. 2) with larger scale changes in the spacer were developed, including a reversed spacer, Gr4Con1, a miss-sense spacer, Gr4Con2, and a double spacer, Gr4Con3. Not surprisingly, the doubled spacer length of Gr4Con3 had no apparent impact on CAH1 pre-protein processing (Supplementary Fig. S3D). The apparent normal processing of construct Gr4Con1 (Fig. 4A), which not only disrupted the junction sites but also reversed the order and orientation of the amino acid sequence of the spacer, indicates the orientation of the spacer does not affect processing of the pre-protein, and this led us to speculate that the physical nature of the spacer may be more important than the amino acid sequence.

The unique construct Gr4Con2, in which the spacer was generated by a frame-shift at the beginning and end of the spacer, has little sequence similarity with the original spacer and disrupts the amino acid sequence at the junction sites as well as most, if not all, identified and unidentified potential endoprotease target sequences within the spacer. The lack of any change in the size of the resulting CAH1 large subunit relative to the positive control Gr1Con1 (Fig. 4A), together with detection and N-terminal sequencing of the small subunit (Table 1), indicates that this spacer with a unique amino acid sequence also does not hinder proteolytic cleavage of the CAH1 pre-protein, thus calling into question whether the nature of the spacer is important for processing or even whether the spacer is required at all.

### Spacer is not required for CAH1 processing

Because the results outlined above indicated that proteolytic cleavage of the CAH1 pre-protein apparently was not solely controlled by the junction sites or by any specific amino acid sequence within the spacer, the role of the spacer was further investigated by a series of large-scale deletions (Group 6; Fig. 2), beginning with complete deletion of the spacer in construct Gr6Con1. Western blot analysis in comparison with the positive control, Gr1Con1, showed a prominent band the same molecular mass as the large subunit of the positive control (37kDa), suggesting proteolytic removal of the small subunit, and a second, less prominent protein band with slightly lower molecular mass (35kDa) (Fig. 4D and Supplementary Fig. S4). The molecular mass of the smaller band is consistent with the expected molecular mass of the CAH1 large subunit lacking glycosylation, as seen with construct Gr5Con2 (see below), although the absence of glycosylation was not confirmed for Gr6Con1. The N-terminal sequence of the upper band from construct Gr6Con1 was consistent with the positive control (Table 1), and the N-terminal sequence of the small subunit obtained after affinity purification confirmed the proteolytic cleavage of the small subunit from the CAH1 pre-protein (Table 1). MALDI-TOF analysis of the more prominent upper band of affinity purified CAH1 of Gr6Con1 failed to detect any peaks matching peptides from the small subunit, further confirming that the small subunit was proteolytically separated from the pre-protein (Supplementary Fig. S5).

Non-reducing SDS-PAGE analysis of CAH1 indicates the CAH1 expressed from Gr6Con1 is not only processed but also apparently assembled into a disulfide-linked heterotetrameric complex indistinguishable in size from that of the control Gr1Con1 (Supplementary Fig. S6). The successful processing and assembly of Gr6Con1 unequivocally demonstrates that the sequence of the spacer is not important for processing; in fact, it demonstrates that the spacer itself appears dispensable.

### Deletions in the flanking sequences of the large and small subunits are still processed

If the spacer itself is unnecessary for processing, the controlling elements for processing of the CAH1 pre-protein might be found in the sequences flanking the spacer in the large and small subunits. To investigate the potential role played by these sequences, two deletion constructs in Group 5, Gr5Con1, and Gr5Con2 (Fig. 2), were designed and expressed in Arabidopsis with alterations in these flanking sequences.

Deletions in construct Gr5Con1 of 10 amino acids from the mature N-terminus of the CAH1 small subunit, when expressed in Arabidopsis, resulted in large subunit protein bands indistinguishable in molecular mass on western blots from that of the control, Gr1Con1 (Fig. 4B), indicating that the absence of these flanking amino acids had no effect on the proteolytic processing of the CAH1 pre-protein.

**Fig. 5.**
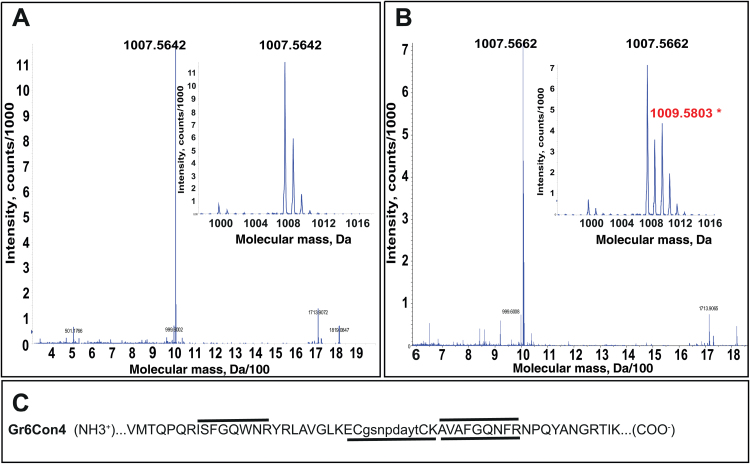
The small subunit fails to be proteolytically cleaved from the large subunit in the Gr6Con4 construct. (A) Mass spectrometry data for the large subunit of Gr5Con2. (B) Mass spectrometry data for Gr6Con4. Molecular mass in daltons is given on the *X*-axis. The inset, smaller box shows peaks between molecular mass 998 and 1016Da from the same mass spectrometry data. Intensity units are given on the *Y*-axis. (C) Partial amino acid sequence of Gr6Con4 spanning large and small subunits. The trypsin digested peptides identified by MALDI-TOF are indicated by the line above the sequence, and the trypsin digested peptides identified by Edman degradation are indicated by the line below the sequence.

On the other hand, deletion in construct Gr5Con2 of the C-terminal 10 amino acids from the CAH1 large subunit resulted in a CAH1 large subunit protein of molecular mass 35kDa, approximately 2kDa smaller than the positive control, Gr1Con1 (37kDa) (Fig. 4C). The 10 amino acids deleted in Gr5Con2 included the asparagine residue (N297) known to be glycosylated (Ishida *et al.*, 1993). N-terminal sequence analysis of the large subunit from Gr5Con2 revealed the expected amino acid sequence (Fig. 2), thus ruling out N-terminal truncation as a possible explanation for the decreased molecular mass. MALDI-TOF analysis of trypsin digested, purified CAH1 large subunit protein from Gr5Con2 failed to identify the C-terminal peptide, but did identify the peptide ISFGQWNR (mass 1007.51Da), which is 10 amino acids upstream from the expected C-terminus (Fig. 5A). No small subunit peptides were identified by MALDI-TOF analysis of the large subunit, and separation of the small subunit was confirmed by N-terminal sequence analysis of the small subunit from affinity purified CAH1 protein (Fig. 2 and Table 1). Again, these results clearly demonstrate the lack of any sole controlling role played by the 10 C-terminal amino acids of the large subunit in the correct proteolytic cleavage of the CAH1 pre-protein.

We developed two additional constructs to test the importance of the disulfide bond-forming cysteine residues in large and small subunits. In Gr5Con3 the cysteine in the small subunit is replaced with alanine, while in Gr5Con4, the cysteine residue in the large subunit is replaced with alanine. Expression of each of these constructs resulted in much reduced levels of protein expression suggesting that formation of disulfide bonds between large and small subunits may be important for stable CAH1 protein expression (Supplementary Fig. S7A, B).

### CAH1 pre-protein processing is compromised by decreasing the number of amino acids separating the disulfide bond-forming cysteines of the large and small subunits

Since the spacer itself was found unnecessary for proteolytic processing of the CAH1 pre-protein, and deletion analysis of the large and small subunit sequences flanking the spacer indicated they also played no controlling role in CAH1 pre-protein processing, we tested the hypothesis that the distance between the intermolecular disulfide bond-forming cysteine residues in the large and small subunits is critical for processing. The total number of amino acids between the disulfide-bond-forming cysteine residues of the small and large subunits in Gr6Con1, in which the entire spacer has been deleted, is 21, so we designed three constructs (Group 6; Fig. 2), Gr6Con4, Gr6Con3 and Gr6Con2, with fewer amino acid residues separating these cysteine residues.

**Fig. 6.**
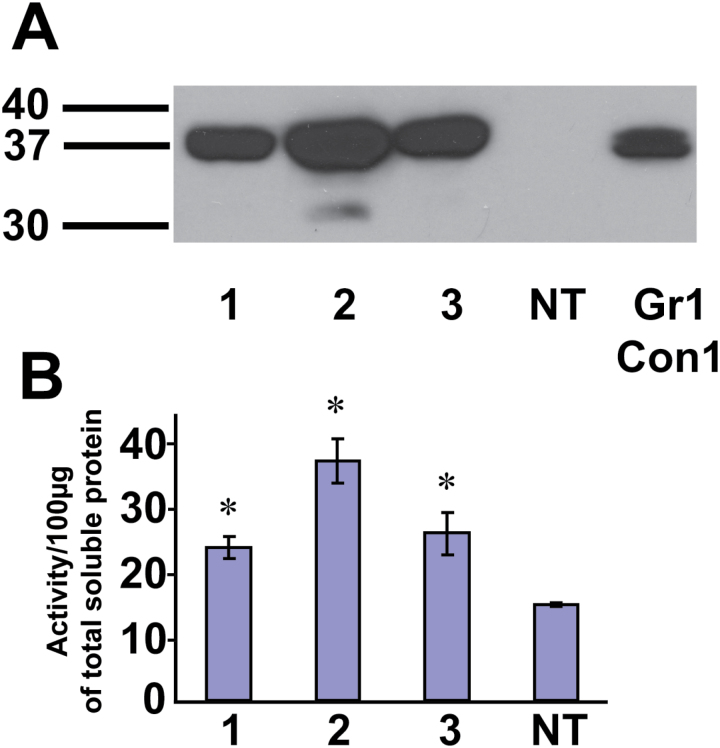
Gr6Con4 construct expresses stable and active CAH1 protein. (A) Western immunoblot of three individual T1 transgenic Arabidopsis plants expressing Gr6Con4, an untransformed control (NT) and a transgenic T1 plant expressing Gr1Con1. Each lane contains 30 µg total soluble protein. (B) Carbonic anhydrase activity for the above-mentioned plants. Total soluble protein was used for the activity assay was 100 µg. Data represented as mean values of three readings with standard error bars. Mean values significantly different from the NT as determined by unadjusted paired *t* tests (*P*<0.05) are denoted by an asterisk.

Construct Gr6Con4 completely lacked the spacer region, 10 amino acids at the C-terminus of the large subunit and four amino acids at the N-terminus of the small subunit, bringing the total number of amino acids between the disulfide-bond-forming cysteine residues to eight, including two amino acids resulting from the addition of a *Bam*HI restriction site. The large subunit of this construct, if processed, should be essentially identical to that of Gr5Con2, which also lacks 10 amino acids at the C-terminus of the large subunit, including the asparagine residue known to be glycosylated (Fig. 2). Western immunoblot analysis (Fig. 4D) showed a molecular mass of 37kDa for the immunodetected CAH1 protein band from Gr6Con4, which is very similar to that of the positive control, Gr1Con1, but significantly higher than the molecular mass of the CAH1 large subunit from Gr5Con2 (35kDa). N-terminal sequence analysis of the affinity purified protein from Gr6Con4 confirmed the removal of the endomembrane targeting signal peptide, confirming that the size difference was not due to lack of removal of the endomembrane targeting signal (Table 1). To determine whether proteolytic removal of the small subunit from the CAH1 pre-protein occurred with this construct, MALDI-TOF analysis was performed on the affinity purified, trypsin digested 37kDa CAH1 protein from Gr6Con4 (Fig. 5B). The data indicate the presence of a peptide (AVAFGQNFR, molecular mass 1009.5803Da) from the normal small subunit retained in the 37kDa protein band, indicating the lack of proteolytic cleavage of the small subunit from the CAH1 pre-protein. Tryptic peptides from the affinity purified 37kDa CAH1 protein of Gr6Con4 were isolated by HPLC and selectively sequenced, allowing identification of the peptide ECGSNPDAYTCK (mass 1287.4986Da) bridging the connection between the large and small subunit sequences, as well as the CAH1 small subunit peptide AVAFGQNFR (mass 1009.13Da), confirming that the mature CAH1 protein from Gr6Con4 retained the small subunit covalently connected to the large subunit. Although unprocessed, the carbonic anhydrase activity assay demonstrated the CAH1 protein from Gr6Con4 to be active (Fig. 6B).

Construct Gr6Con3 lacks the spacer region and 10 amino acids from the C-terminus of the large subunit (Fig. 2), but the amino acid sequence of the small subunit is intact, so the total number of amino acids between the disulfide-bond-forming cysteine residues is 12, including two amino acids from the addition of a *Bam*H1 restriction site. Western blot analysis comparing the molecular mass of the immunodetected CAH1 protein from Gr6Con3 with that from the positive control, Gr1Con1, as well as from Gr5Con2, revealed two consistent, clear CAH1 protein bands (Fig. 4D), with the upper, more intense band (molecular mass 37kDa) similar to the molecular mass of both the positive control and Gr6Con4. Also identified was a lower, less intense band (molecular mass 35kDa) similar to the molecular mass of CAH1 from Gr5Con2. The N-terminal sequences of the two protein bands from affinity purified Gr6Con3 CAH1 were consistent with the N-terminus of the large subunit of Gr1Con1 (positive control), indicating successful and normal cleavage of the endomembrane targeting signal peptide (Table 1). Protein samples from both protein bands (molecular mass 35 and 37kDa) were also subjected to trypsin digestion and MALDI-TOF analysis, which indicated the presence of small subunit peptides from the upper band, but not from the lower band (Supplementary Fig. S8A, B). Tryptic peptides from the upper band of Gr6Con3 CAH1 protein were resolved by HPLC and selectively sequenced, revealing the presence of tryptic peptides with masses consistent with the peptide ECGSAESANPDAYTCK (mass 1645.64Da) bridging between the large and small subunit sequences, as well as the CAH1 small subunit peptides NPQYANGR (mass 919.43Da), confirming that the upper band of Gr6Con3 included the small subunit covalently bonded with the large subunit of CAH1. Furthermore, although staining was not sensitive enough to detect a small subunit band, tryptic digestion and MALDI-TOF analysis of proteins in the expected small subunit size range identified a peptide of the expected mass (1009.4257Da) of the small subunit peptide AVAFGQNFR (Supplementary Fig. S8C). Therefore, these results demonstrate partial processing of CAH1, where the small submit was processed from only some of the CAH1 pre-protein molecules, while others remained unprocessed.

Construct Gr6Con2 lacks the spacer and nine N-terminal amino acids in the flanking region of the small subunit (Fig. 2) but contains an intact large subunit, so 16 amino acids separate the disulfide-bond-forming cysteine residues of the small and large subunits. Western blot analysis consistently indicated the presence of four separate immunodetectable CAH1 protein bands. Relative to the molecular mass of the CAH1 large subunit from the positive control, Gr1Con1 (37kDa), the upper two bands appeared slightly larger (42 and 40kDa, respectively), the second lowest band appeared similar in size (37kDa), and the lowest band appeared slightly smaller (34kDa), similar to that from Gr5Con2 (35kDa) (Fig. 4D and Supplementary Fig. S3). Affinity purified CAH1 protein was subjected to N-terminal sequencing of the large subunits, but due to our inability to resolve all four bands sufficiently, the top two bands and the lower two bands were analysed as pairs. The large subunit N-terminal sequences, including minor peptide sequences, were all consistent with the positive control, indicating that all four bands had same N-terminal sequence (Fig. 2). MALDI-TOF analysis of the top two bands together, again due to our inability to resolve them sufficiently, detected peptides from the small subunit, indicating the lack of proteolytic separation of the small subunit in at least one of the two bands, if not both (Supplementary Fig. S9A). Small subunit peptides were not detected by MALDI-TOF analysis in either of the lower two bands analysed separately, indicating the successful proteolytic removal of the small subunit in both (Supplementary Fig. S9B, C). Tryptic peptides from the upper two bands together were resolved by HPLC and selectively sequenced, revealing the presence of small subunit peptides AVAFGQNFR (mass 1009.13Da) and NPQYANGR (mass 919.43Da), and confirming that at least one of the two larger bands resulted from unprocessed CAH1 protein where the small subunit was still covalently bonded with the large subunit. The successful detection of a small subunit in affinity purified CAH1 from Gr6Con2, confirmed by N-terminal sequence analysis (Fig. 2), is consistent with the lower two immunodetectable bands representing proteolytically processed CAH1 containing only the large subunit. As with Gr6Con3, these results demonstrate partial processing of CAH1 in Gr6Con2, where the small submit was processed from only some of the CAH1 pre-protein molecules, while others remained unprocessed.

Regardless of whether the CAH1 from Gr6Con1, Gr6Con2 and Gr6Con3 was only partially processed, only one band was detected for each on non-reducing SDS-PAGE (Supplementary Fig. S6), suggesting that the processed and unprocessed peptides assembled into disulfide-linked complexes indistinguishable in size from that of the control Gr1Con1 CAH1.

## Discussion

Site-specific, limited proteolysis is an important step that converts an inactive pre-protein into a functional, correctly folded mature protein. Investigation of endoproteases involved in pro-protein conversion has been extensively studied for over a decade. Various proteins trafficking through the endomembrane system that undergo limited, site-specific proteolysis have been studied in detail in mammalian cells (Seidah, 2011; Seidah *et al.*, 2013) as well as in plants (Murphy *et al.*, 2012). In plants, important roles played by post-translational modification, including site-specific proteolysis, especially in generation of biologically active forms of small, secreted peptides, have been well documented (Matsubayashi, 2011). Even though in some cases evidence indicates the involvement of dibasic residue targeting subtilisin-like proteases (Srivastava *et al.*, 2008; Wolf *et al.*, 2009), in many cases a clear understanding of the proteolytic enzymes involved is still lacking (Jiang and Rogers, 1999; Francois *et al.*, 2002; Qiao *et al.*, 2012; Gu *et al.*, 2012; Sugawara, *et al.*, 2013).

Chlamydomonas CAH1 is proteolytically processed from a single pre-protein to release both large and small subunits that assemble into a heterotetramer (Kamo *et al.*, 1990), and this processing appears to be conserved in higher plants (Roberts and Spalding, 1995). Similar proteolysis also has been suggested by Francois, *et al.* (2002) in Arabidopsis during transgenic expression of a polyprotein construct leading to production of two different antimicrobial proteins separated by a spacer-like linker peptide. In the processing of these antimicrobial proteins the initial proteolytic cleavage was suggested to be dependent on occurrence of a diacidic site such as Asp/Glu, but no direct evidence was provided to support the involvement of or requirement for the diacidic residues (Tailor *et al.*, 1997; Francois *et al.*, 2002). More recently similar proteolysis has also been suggested in the maturation of a small, secreted, growth promoting peptide, phytosulfokines (PSKs) as well as of pectin methylesterase (PME) (Srivastava *et al.*, 2008; Wolf *et al.*, 2009). In both cases, dibasic amino acid targeting subtilisin-like serine endopeptidases were shown to control the proteolysis. Based on earlier reports, we originally hypothesized that CAH1 maturation would involve limited and specific proteolysis through protease recognition of one or more specific amino acid sequences in the pre-protein. Since we envisioned using this pre-protein or poly-protein processing system in Arabidopsis for coordinated expression of multiple protein coding sequences separated by the CAH1 spacer, we first proceeded to identify the factors determining the spacer’s proteolytic removal. Based on that motivation, we designed a hierarchical series of constructs intended to identify and pinpoint the target site(s) for proteases responsible for the proteolytic maturation of the CAH1 pre-protein.

The first set of constructs (Group 1) disrupted or modified the previously demonstrated junction sites between the spacer and the large and small subunits. The second set (Group 2 and Group 3) eliminated identifiable, potential protease target sites within the spacer by sequentially deleting large stretches of the spacer. The failure of any of the modifications from Groups 1, 2 and 3 to disrupt the normal processing of CAH1 suggested the lack of any specific amino acid recognition sequence controlling its proteolytic processing. The failure of constructs Gr4Con1 and Gr4Con2 containing, respectively, a reversed spacer amino acid sequence and a unique amino acid spacer sequence intended to disrupt spacer processing, further confirmed the lack of specific amino acid sequences as essential specific protease recognition sites in the spacer region.

The failure to identify a specific targeting sequence essential for proteolytic processing within the spacer prompted us to explore the large and small subunit regions flanking the spacer between the cysteine residues involved in intermolecular disulfide bond formation between these subunits. These cysteines are located nine amino acids upstream of the mature C-terminus in the large subunit and 10 amino acids downstream of the mature N-terminus in the small subunit (Fukuzawa *et al.*, 1990; Kamo *et al.*, 1990). Constructs lacking these specific disulfide bond-forming cysteine residues, Gr5Con5 and Gr5Con6, resulted in much reduced levels of protein expression, indicating the importance of disulfide bond formation between large and small subunits for stable CAH1 protein expression (Supplementary Fig. S7A, B).

Since the peptide between these cysteine residues is composed of an unusually high proportion of charged residues (Fig. 1; 17 of the 35 spacer peptide amino acids are charged), we hypothesized that following insertion of the CAH1 pre-protein into the endoplasmic reticulum and proteolytic processing of the N-terminal signal peptide, the peptide sequences representing the mature large and small subunits may fold into their respective tertiary structures, leaving the connecting hydrophilic spacer peptide as a loop extending into the solvent. The published crystal structure of CAH1 (Suzuki *et al.*, 2011) supports our hypothesis. The crystal structure of the mature heterotetramer shows that the small subunit is embedded deep in the pocket that forms an active site holding the catalytic Zn ion. The structure emphasizes a protruding, unfolded spacer region that is presumably accessible to proteases, potentially making the proteolysis of the spacer an opportunistic but non-specific process. After opportunistic cleavage of the spacer by one or more specific or non-specific endoproteases, aminopeptidases and carboxypeptidases might progressively cleave exposed residues until steric hindrance from the folded large and small subunits prevents them from progressing further.

We tested this hypothesis with a series of constructs incorporating deletions and modifications in the sequences of the large and small subunits flanking the spacer, as well as constructs with a progressively reduced number of amino acids between the disulfide-bond-forming cysteine residues of the large and small subunits. Our goal was to re-confirm that no specific sequence in the spacer or flanking sequences was required for processing and to reduce the number of amino acids between the disulfide-bond-forming cysteine residues, thereby progressively decreasing the size of the hypothetical spacer loop extending into the solvent and potentially inhibiting proteases from gaining access.

Because no apparent effect on CAH1 processing was noted with deletions only in the flanking sequences of the large and small subunits, we concluded that no unique role in processing is played by these flanking sequences. The only effect we noted with these constructs was a decrease in the molecular mass of the large subunit from construct Gr5Con2, which lacks all nine amino acids at the C-terminus of the large subunit, including a confirmed N-linked glycosylation site. Thus the decreased large subunit molecular mass for Gr5Con2 can be attributed to the missing N-linked glycosylation and lack of a potentially bulky sugar complex.

The results from construct Gr6Con1, which entirely lacks the spacer amino acid sequence, provided an important advance in our understanding of the processing of the CAH1 pre-protein, in that it demonstrated clearly that the spacer *per se* was not needed for processing or assembly. Together with the lack of any effect of the flanking sequences of the large and small subunits, this observation suggested that the length and physical characteristics of the amino acid stretch between the disulfide-bond-forming cysteines of the large and small subunits might be the key to processing.

Four constructs, Gr6Con1, Gr6Con2, Gr6Con3 and Gr6Con4, representing a progressively decreasing number, from 21 (Gr6Con1), to 17 (Gr6Con2), to 12 (Gr6Con3), to eight (Gr6Con4) amino acids between the key cysteine residues, were used to test the hypothesis that the number of amino acids between these cysteine residues is key to allowing an opportunistic processing of the CAH1 pre-protein. As mentioned above, our observations indicate Gr6Con1, with 21 amino acids separating the key cysteines, was processed normally. Although there was, to a variable extent, a slightly lower molecular mass presumptive large subunit detected that may represent either a truncated or a non-glycosylated large subunit, there was no evidence from the MALDI-TOF analysis for the presence of a non-processed CAH1 protein.

On the other hand, Gr6Con4, with only eight amino acids separating the key cysteines, showed no evidence of processing at all, other than removal of the N-terminal signal peptide. This conclusion is supported by the presence of a single band with an apparent molecular mass much higher than the large subunit of Gr5Con2, with which Gr6Con4 shares in lacking the whole large subunit flanking sequence, including the asparagine residue glycosylated in the control. Confirmation of the lack of processing was provided by the presence of small subunit sequences, confirmed by N-terminal sequencing of tryptic peptides, detected in the MALDI-TOF analysis of the purified, single CAH1 protein band from Gr6Con4, including confirmation of the peptide bridging the large and small subunit sequences. Enzyme activity assays performed using total protein extract from three separate transgenic Gr6Con4 T1 generation plants indicated an active form of the CAH1 enzyme, suggesting proteolytic separation of the small subunit is not necessary for enzyme activity.

Constructs Gr6Con3 and Gr6Con2, with 12 and 17 amino acids, respectively, separating the key cysteine residues, both yielded multiple large-subunit-containing CAH1 protein bands, which were confirmed to represent partial processing of the pre-proteins produced from these constructs. The two distinct bands observed with Gr6Con3 were demonstrated to represent an unprocessed pre-protein and a processed large subunit of CAH1 in the larger and smaller bands, respectively. Similarly, the four distinct bands observed with Gr6Con2 were demonstrated to represent partial processing of the pre-protein, with the two upper bands representing unprocessed pre-protein and the two lower bands representing processed large subunits. The best explanation for the occurrence of these four large-subunit-containing CAH1 protein bands resulting from expression of the Gr6Con2 construct appears to be that in both the upper, unprocessed pair and the lower, processed pair, the lighter of each pair either has altered glycosylation or is lacking glycosylation completely, presumably of the asparagine in the large subunit flanking region. Therefore, the results from constructs Gr6Con1, Gr6Con2, Gr6Con3 and Gr6Con4 demonstrate that decreasing the number of amino acids between the disulfide-bond-forming cysteines of the large and small subunits below about 21 results in a decrease in the efficiency of CAH1 pre-protein processing, resulting in a complete lack of processing when only about eight amino acids separate the cysteines.

Our results suggest that the previously identified CAH1 spacer region is not necessary for the proteolytic separation of the large and small subunits, although it may play an important role in the correct post-translational modification of the protein, such as N-glycosylation. We can also conclude that the efficiency of the proteolytic machinery in cleaving the small subunit from the CAH1 pre-protein increases with the number of amino acids separating the disulfide-bond-forming cysteine residues, at least in the range of 8–21 amino acids. Our results also indicate that proteolytic separation of the small subunit is not a necessity for the production of an active enzyme, since the unprocessed mature protein from Gr6Con4 was active. This result raises interesting questions about the evolutionary and functional significance of the proteolytic removal of the spacer. Further experimentation using a homologous system will be required to reveal the importance of the proteolytic events in the context of proper localization, optimum post-translational modification or optimum activity of the mature CAH1.

This research reveals a potentially unique proteolytic processing progression. Our results suggest that proteolytic processing of CAH1, albeit in a heterologous system, is an opportunistic process that relies on an unidentified endoprotease/s to make an initial, perhaps non-specific, cut in the hydrophilic amino acid sequence between the disulfide-bond-forming cysteine residues of the large and small subunits, the efficiency of which is related to the physical distance between these cysteine residues. The variable N-terminal sequence results from the small subunit suggests that after the initial cut, the exposed residues are cleaved by aminopeptidases and carboxypeptidases until the steric hindrance of the folded large and small subunits prevent them from cleaving any further. Our inability to pinpoint a requirement of any specific amino acid sequence for the proteolysis suggests involvement of unknown, probably multiple, opportunistic endoproteases.

## Supplementary data

Supplementary data are available at *JXB* online.

Figure S1. Schematic representation for *in planta* CAH1 expression cassette.

Figure S2. Arabidopsis expresses processed and active CAH1.

Figure S3. Spacer does not seem to contain any specific motif that controls proteolytic cleavage of the small subunit.

Figure S4. CAH1 pre-protein processing is compromised by decreasing the number of amino acids separating the disulfide bond forming cysteines of the large and small subunits.

Figure S5. Spacer is not required for the proteolytic processing of the CAH1 pre-protein.

Figure S6. Group six constructs form stable heterotetramers.

Figure S7. Mutants with mutated disulfide bond forming cysteine residues show weak protein expression with an altered large subunit size.

Figure S8. Gr6Con3 is partially processed.

Figure S9. Gr6Con2 is partially processed.

Supplementary materials and methods. Development of constructs explained in detail with primer sequences.

Supplementary Data
