# Supplementary material for: Opportunistic proteolytic processing of carbonic anhydrase 1 from Chlamydomonas in Arabidopsis reveals a novel route for protein maturation
Source: J Exp Bot. 2016 Feb 24;67(8):2339–51. doi: 10.1093/jxb/erw044 (PMC4809292; doi:10.1093/jxb/erw044)
Supplement: Supplementary Data [file supp_67_8_2339__index.html]

Opportunistic proteolytic processing of carbonic anhydrase 1 from Chlamydomonas in Arabidopsis reveals a novel route for protein maturation — Opportunistic proteolytic processing of carbonic anhydrase 1 from Chlamydomonas in Arabidopsis reveals a novel route for protein maturation — Supplementary Data 

# Opportunistic proteolytic processing of carbonic anhydrase 1 from Chlamydomonas in Arabidopsis reveals a novel route for protein maturation

## Supplementary Data

Data files

- supplementary\_figures\_S1\_S9\_methods.pdf - Supplementary Data
