# Supplementary material for: Opportunistic proteolytic processing of carbonic anhydrase 1 from Chlamydomonas in Arabidopsis reveals a novel route for protein maturation
Source: J Exp Bot. 2016 Feb 24;67(8):2339–51. doi: 10.1093/jxb/erw044 (PMC4809292; doi:10.1093/jxb/erw044)
Supplement: Supplementary Data [file supp_erw044_supplementary_figures_S1_S9_methods.pdf]

Opportunistic proteolytic processing of Carbonic Anhydrase 1 from Chlamydomonas in Arabidopsis reveals a novel route for protein maturation.

Parijat S. Juvale, Ryan L. Wagner, and Martin H. Spalding

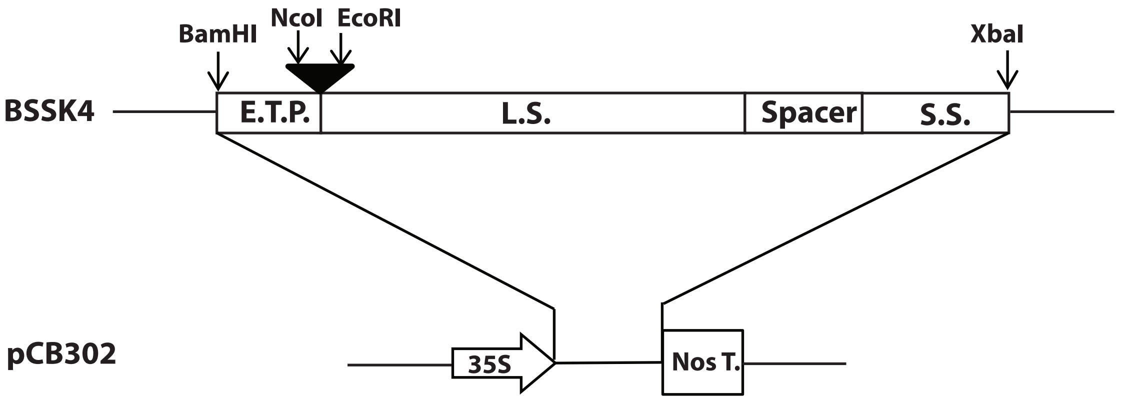

Figure S1. Schematic representation for in planta CAH1 expression cassette.
Schematic representation of the expression cassette for the expression of our constructs in the BSSK4 vector, above, and the binary vector pCB302 below. E.T.P. is endomembrane targeting peptide coding sequence. L.S. is large subunit and S.S. is small subunit.

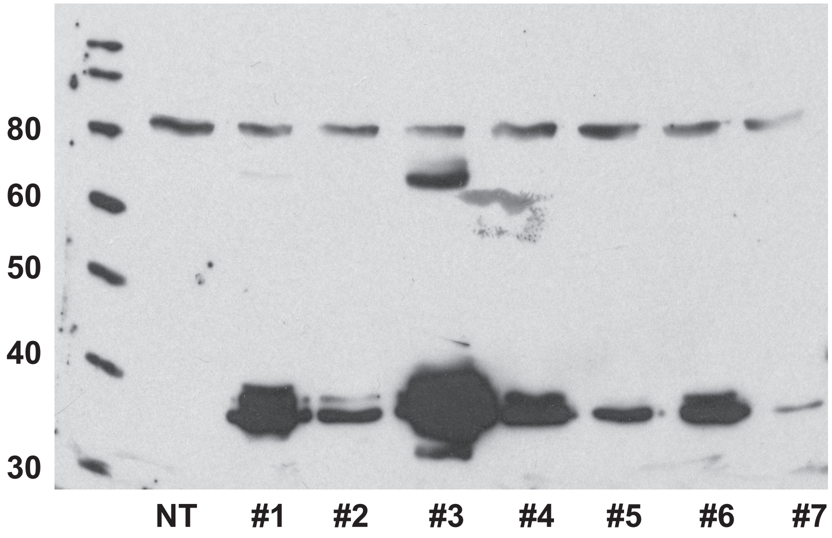

Figure S2. Arabidopsis expresses processed and active CAH1.
Western immunoblot of 7 individual T1 transgenic Arabidopsis plants expressing Gr1Con1 and an untransformed control (NT). All transgenic lines show apparent double bands for CAH1, apparently due to differential glycosylation.
Each lane contains 30 µg total soluble protein.

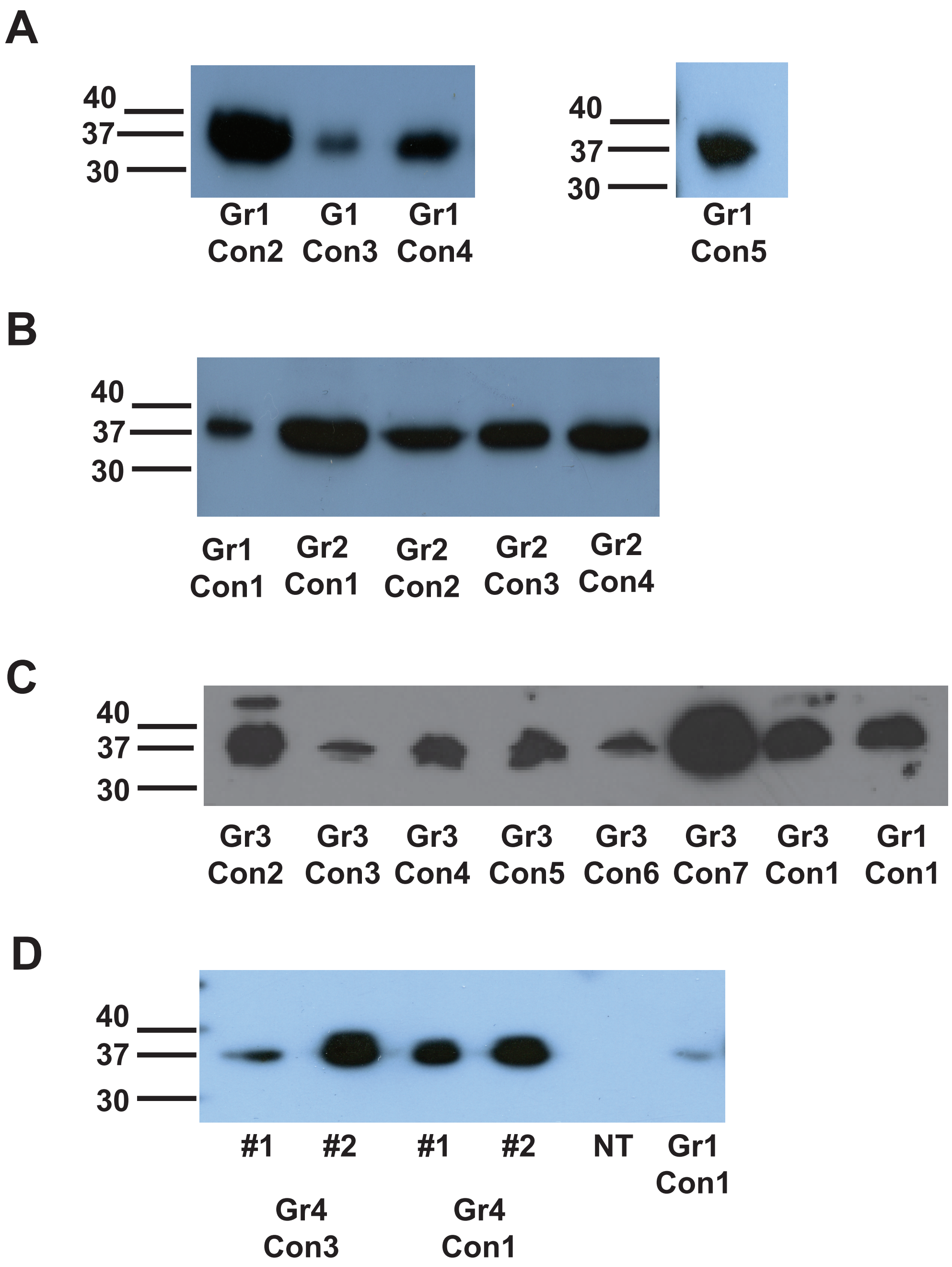

Figure S3. Spacer does not seem to contain any specific motif that controls proteolytic cleavage of the small subunit.

A. Western immunoblot analysis of transgenic Arabidopsis plants expressing group 1 constructs.

B. Western immunoblot analysis for size comparison between the large subunits of the group 2 constructs.
Each lane contains 10 µg total soluble protein.

C. Western immunoblot analysis for size comparison between the large subunits of the group 3 constructs.
Each lane contains 10 µg total soluble protein.

D. Western immunoblot analysis for size comparison between the large subunits of the group 4 constructs.
Each lane contains 10 µg total soluble protein. Two indepedent transgnic plants from Gr4Con3 and Gr4Con1 constructs are used.

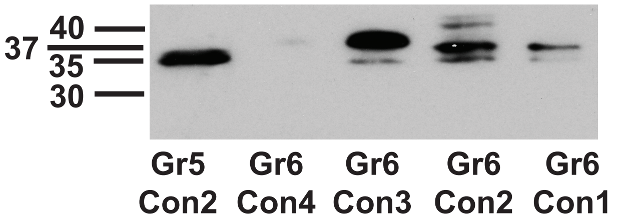

Figure S4. **CAH1 pre-protein processing is compromised by decreasing the number of amino acids separating the disulfide bond forming cysteines of the large and small subunits.**

Western immunoblot analysis for size comparison between the large subunits of the group 6 constructs and Gr5Con2. Amount of proteins added in each lane are as follows; Gr5Con2: 28 µg, Gr6Con4: 2 µg, Gr6Con3: 40 µg, Gr6Con2: 36 µg, Gr6Con1: 15 µg.

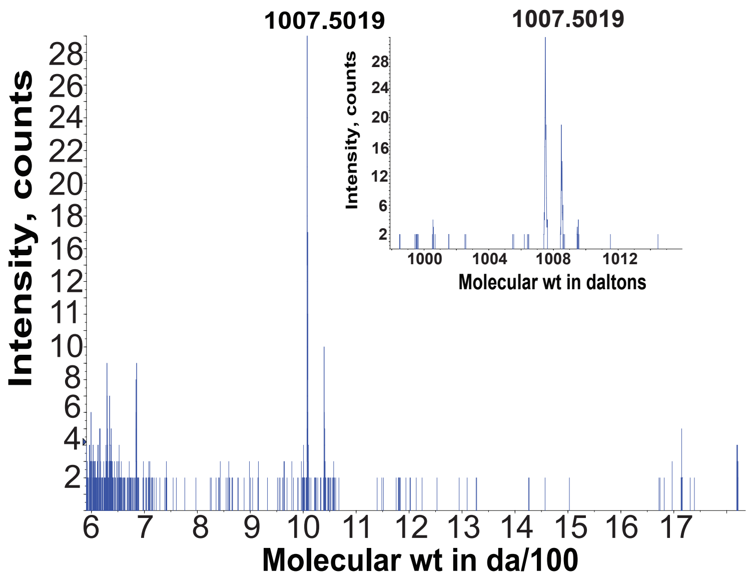

Figure S5. Spacer is not required for the proteolytic processing of the CAH1 pre-protein.

Mass spectrometry data of the large subunit of Gr6Con1. Molecular weight in daltons is given on the X axis. The inset, smaller box shows peaks between molecular weight 998 daltons to 1016 daltons from the same mass spectrometry data. Intensity units are given on the Y axis.

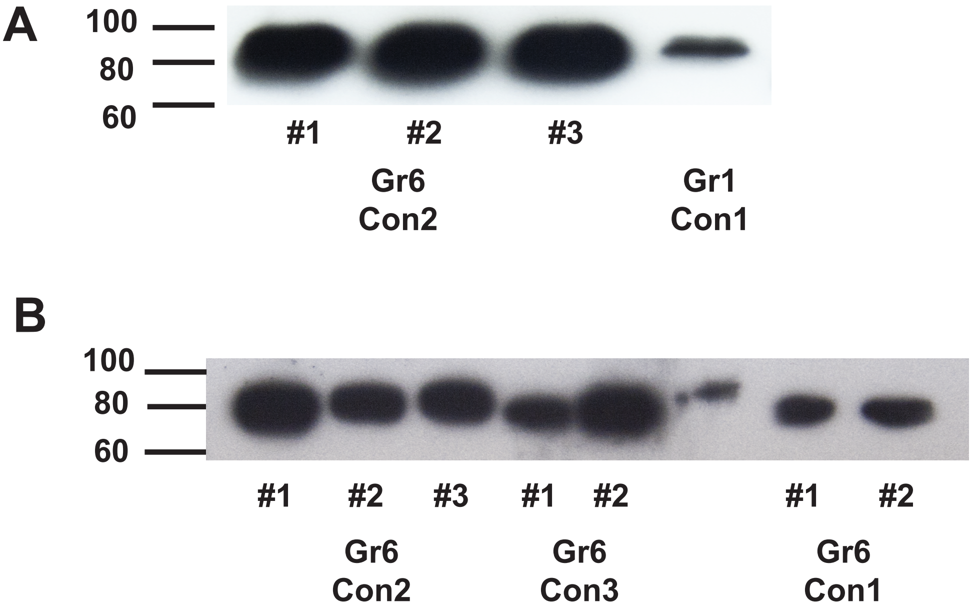

Figure S6. Group six constructs form stable heterotetramers.

A & B Western immunoblot analysis of total soluble protein from independent transgenic plants expressing group six constructs with intact disulfide bonds. 10 µgm of total soluble protein in each lane.

A

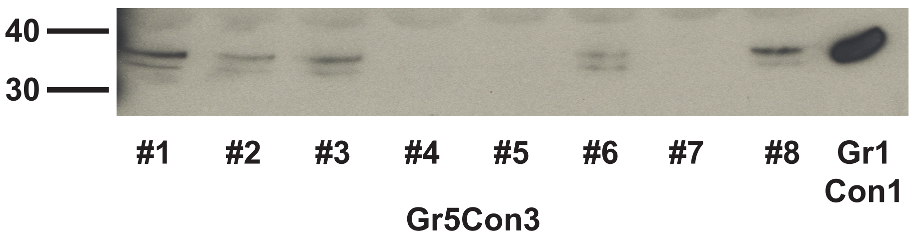

B

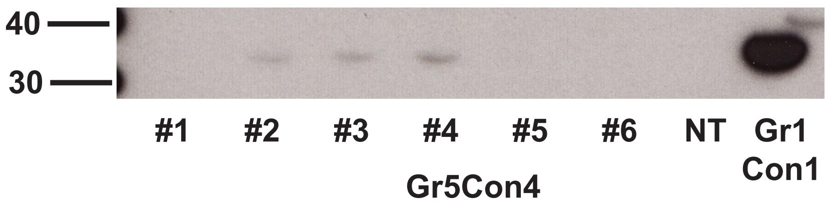

Figure S7. Mutants with mutated disulfide bond forming cystine residues show weak protein expression with an altered large subunit size.

A. Western immunoblot analysis of transgenic Arabidopsis plants expressing Gr5Con3 construct.

B. Western immunoblot analysis of transgenic Arabidopsis plants expressing Gr5Con4 construct.

Each lane contains 10 µg total soluble protein.

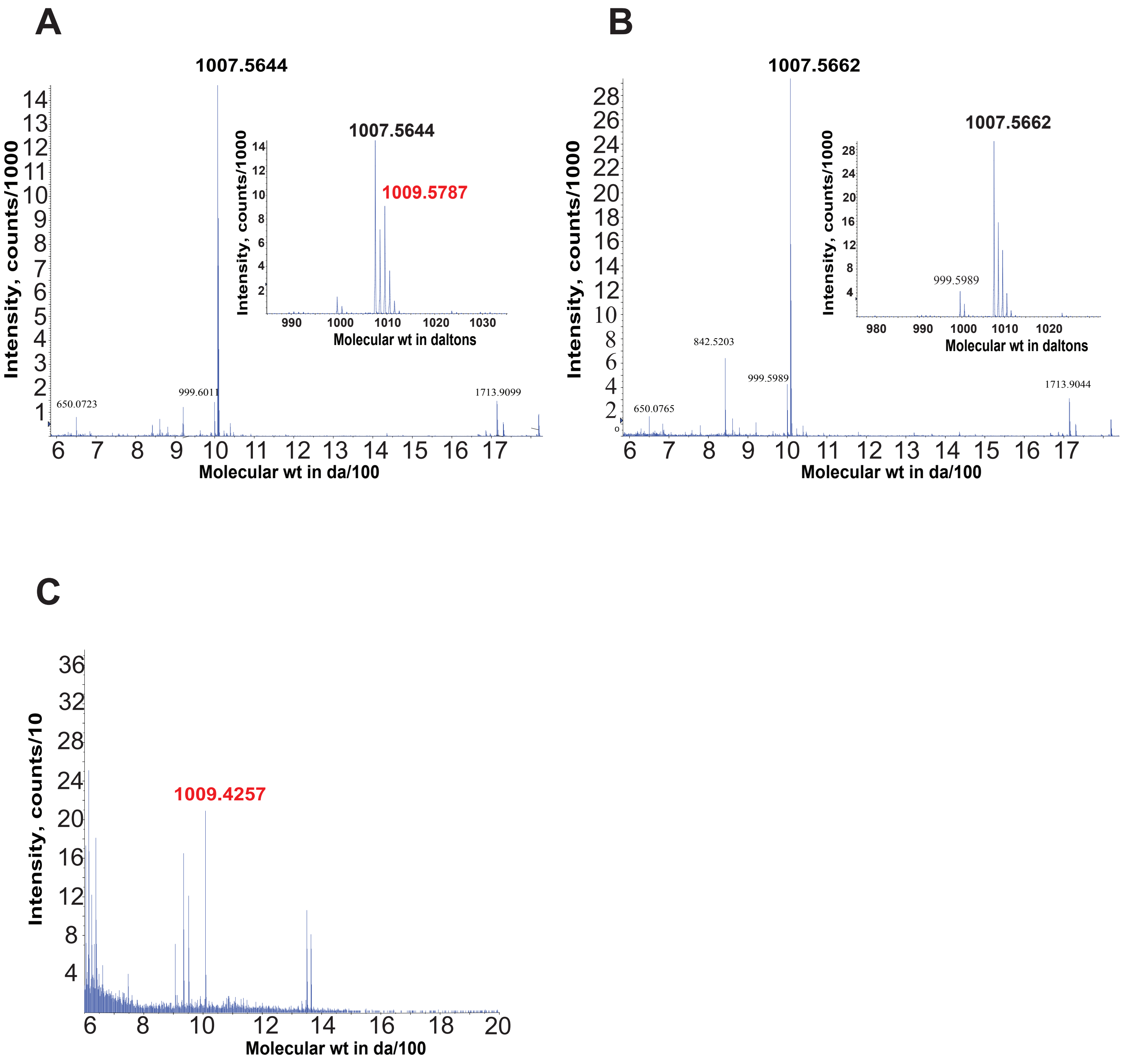

D

Gr6Con3 (NH_3^+)...HVMTQPQRISFGQWNRYRLAVGLKECgsaesanpdaytCKAVAFGQNFRNPQYANGRTIKLARYH...(COO^-)

Figure S8. Gr6Con3 is partially processed.

A. Mass spectrometry data of the upper band of Gr6Con3.

B. Mass spectrometry data of the lower band of Gr6Con3.

C. Mass spectrometry data of the small subunit sequences of GrCon3.
Molecular weight in daltons is given on the X axis.
The inset, smaller box shows peaks between molecular weight 980 daltons to 1030 daltons from the same mass spectrometry data. Intensity units are given on the Y axis.

D. Partial amino acid sequence of Gr6Con3 spanning large and small subunits.
The trypsin digested peptides identified by MALDI-TOF are indicated by the line above and ones identified by N terminal sequence analysis are indicated by the line below the sequence.

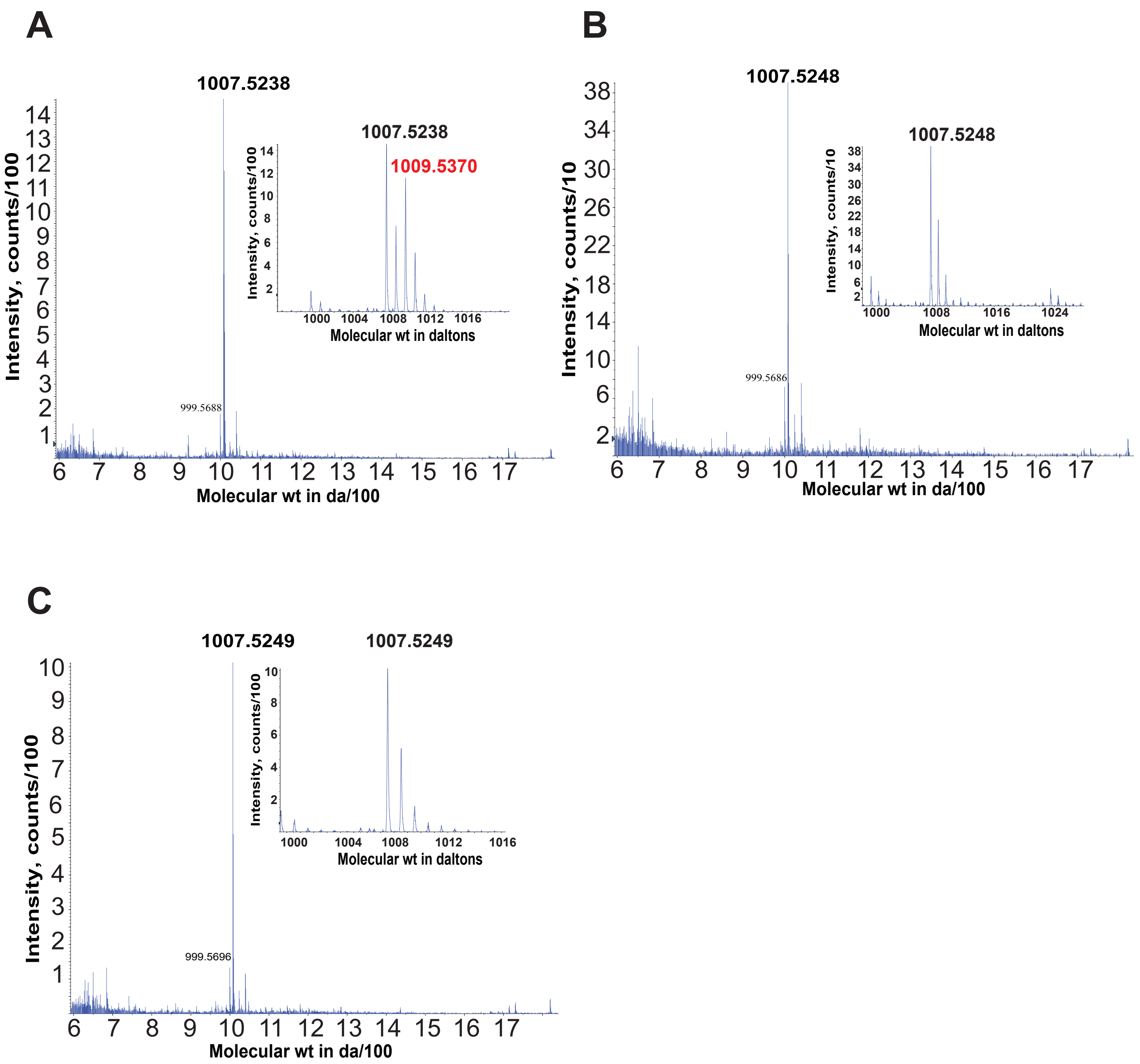

D

Gr6Con2 (NH3$^+$)...TQPQRISFGQWNRYRLAVGLKECnstetaadagsnpdaytCKAVAFGQNFRNPQYANGRTIKLARYH...(COO$^-$)

Figure S9. Gr6Con2 is partially processed.

A. Mass spectrometry data of the upper two bands of Gr6Con2.

B. Mass spectrometry data of the third band (from top) of Gr6Con2.

C. Mass spectrometry data of the forth band (from top) of GrCon2.
Molecular weight in daltons is given on the X axis. The inset, smaller box shows peaks between molecular weight 990 daltons to 1030 daltons from the same mass spectrometry data. Intensity units are given on the Y axis.

D. Partial amino acid sequence of Gr6Con2 spanning large and small subunits.
The trypsin digested peptides identified by MALDI-TOF are indicated by the line above and ones identified by N terminal sequence analysis are indicated by the line below the sequence.

Supplemental Materials and Methods.

Group 1 and group 2 constructs:

To generate constructs in group 1 and 2, Gr1Con1 in BSSK4 vector was used as a template. For each construct forward and reverse primers were designed (sequences given below) and standard PCR methodology was used in order to generate mutant version of *CAH1* with desired amino acid sequence. The resultant construct in vector BSSK4 was sequenced using T7-2 and T3 sequencing primers at Iowa State university (ISU) DNA facility.

Gr1Con2

Forward 5' ACGGAGACCGCCGGGGACGGGGGCCACCACCAC 3'

Reverse 5' GTGGTGGTGGCCCCCGTCCCCGGCGGTCTCCGT 3'

Gr1Con3

Forward 5' GAGACCGCCGCGGGCGGCGGCCACCACCAC 3'

Reverse 5' GTGGTGGTGGCCGCCGCCCGCGGCGGTCTC 3'

Gr1Con4

Forward 5' CACTACTTCCGCCGCGGAGGTGGAGGTGAGTCCGCGAACCCC 3'

Reverse 5' GGGGTTCGCGGACTCACCTCCACCTCCGCGGCGGAAGTAGTG 3'

Gr1Con5

First two primers were used to develop a *CAH1* construct in BSSK4 vector to introduce mutations at the junction of the large subunit and the spacer.

Forward 5' GAGACCGCCGCGGACCACCACCACCACCACCACCAC 3'

Reverse 5' GTGGTGGTGGTGGTGGTGGTGGTCCGCGGCGGTCTC 3'

The resultant construct in BSSK4 was used as a template and following primers were used to develop the final *CAH1* construct with desired mutations at the junction of spacer and small subunit.

Forward 5’ CACTACTTCCGCCGCGTGATGGGAGGTGAGTCCGCGAACCCC 3’

Reverse 5’ GGGGTTCGCGGACTCACCTCCCATCACGCGGCGGAAGTAGTG 3’

Gr2Con1

Forward

5’GACGCCGGCCACCACCACCACCACGCCGCCCTGCTGCACAACCACGCGC

ACCTG 3’

Reverse

5’CAGGTGCGCGTGGTTGTGCAGCAGGGCGGCGTGGTGGTGGTGGTGGCCGGC

GTC 3’

Gr2Con2

Forward 5’

ACCTCCGAGCCCAAGCACTACTTCGCCGCCGTGATGCTGGCCGAGTCCGCGAAC 3’

Reverse 5’

GTTCGCGGACTCGGCCAGCATCACGGCGGCGAAGTAGTGCTTGGGCTCGGAGGT 3’

Gr2Con3 was generated by using Gr1Con2 primers on a BSSK4 vector Gr1Con1 as a template.

Gr2Con4

Forward 5’ CACCACCGCCGCCTGCTGGCACACGCGCACCTGGAGGAGGTG 3’

Reverse 5’ CACCTCCTCCAGGTGCGCGTGTGCCAGCAGGCGGCGGTGGTG 3’

Group 3 constructs:

Gr3Con1 and Gr3Con2 constructs:

A DNA fragment containing the ER targeting sequence and the large subunit of *CAH1* was PCR amplified with a BamH1 restriction site at the 3' end from Gr1Con1 using the forward primer T7-2 (5' CGACTCACTATAGGGCGAAT 3') and the reverse primer (5' TTGGATCCGGCGTCCGCGGT 3'). This amplified sequence was digested with the restriction enzymes, Nco1 and BamH1 to generate an oligonucleotide coding for large subunit only. The second sequence, containing a truncated portion of the spacer and the *CAH1* small subunit was amplified using the forward primer (5' TTGGATCCCGCCGCCTGCTGCAC 3') for Gr3Con1 and (5' AAAGGATCCGAGGAGGTGCCTGCC 3') for Gr3Con2 , and the reverse primer T3-2 (5' GCTATGACCATGATTACGCC 3') with a BamH1 restriction site on the 5' end. This amplified sequence was digested with the restriction enzymes, Bamh1 and Xba1. The Gr1Con1 was digested with Nco1 and Xba1 to generate a digested vector containing the ER targeting sequence only. All three pieces were ligated together to produce the final product, a modified *CAH1* with the restriction site BamH1 inserted between the truncated spacer and the large subunit coding segment.

Gr3Con3 to Gr3Con6:

A DNA fragment containing the ER targeting sequence and the large subunit of *CAH1* was PCR amplified Hpa1 at 5' end from Gr1Con1 using the forward primer T7-2 (5' CGACTCACTATAGGGCGAAT 3') and the reverse primer LSLPHP (5' TTTGTTAACGGCGTCCGCGGCGGT 3'). The amplified product was digested with the restriction enzymes, BamH1 and Hpa1. The CAH1 small subunit was amplified using primers SUUP2 (5' ACTAGTGCCGAGTCCGCGAAC 3') and T3-2(5'

GCTATGACCATGATTACGCC 3'), and was digested with the restriction enzymes, Spe1 and Xba1. The following nucleotide sequences were obtained from Integrated DNA Technologies, Inc.

Gr3Con3:-

Forward 5' AAC TACTTCCGCCGCGTGATGCTGA 3'

Reverse 5' CTAGTCAGCATCACGCGGCGGAAGTAGTT 3'

Gr3con4:-

Forward 5' AACGGCCACCACCACCACCACCGCCGCA 3'

Reverse 5' CTAGTGCGGCGGTGGTGGTGGTGGTGGCCGTT 3'

Gr3Con5:-

Forward 5' AACGAGGAGGTGCCTGCCGCCA 3

Reverse 5' CTAGTGGCGGCAGGCACCTCCTCGTT 3'

Gr3con6:-

Forward 5' AACCTGCTGCACAACCACGC 3'

Reverse 5' CTAGTCGCGTGGTTGTGCAGCAGGTT 3'

For individual constructs, the two corresponding oligonucleotides were mixed in equal proportions. The resultant double stranded oligonucleotide had a digested Hpa1 restriction site on 5' end and a digested Spe1 site on the 3' end. These double stranded oligonucleotides were ligated with the Hpa1-Spe1 digested Gr4Con1 construct (see below) in BSSK4 vector.

Gr3Con7:

A DNA fragment containing the ER targeting sequence and the large subunit of *CAH1* was PCR amplified with a BamH1 restriction site at the 3' end from Gr1Con1 using the forward primer T7-2 (5' CGACTCACTATAGGGCGAAT 3') and the reverse primer (5'

AAGGATCCCAGGTGCGCGTGGTT 3’). This amplified sequence was digested with the restriction enzymes, Nco1 and BamH1 to generate an oligonucleotide coding for large subunit and truncated spacer. The second sequence the *CAH1* small subunit was amplified using the forward primer (5’ AAGGATCCGCCGAGTCCGCGAAC 3’) and the reverse primer T3-2 (5’ GCTATGACCATGATTACGCC 3’) with a BamH1 restriction site on the 5’ end. This amplified sequence was digested with the restriction enzymes, Bamh1 and Xba1. Gr1Con1 was digested with Nco1 and Xba1 to generate a digested vector containing the ER targeting sequence only. All three pieces were ligated together to produce the final product, a modified *CAH1* with the restriction site BamH1 inserted between the truncated spacer and the small subunit coding segment.

Group 4 constructs:

Gr4Con1:

A DNA fragment containing the ER targeting sequence and the large subunit of *CAH1* was PCR amplified with a Hpa1 at 3’ end from Gr1Con1 using the forward primer T7-2 (5’ CGACTCACTATAGGGCGAAT 3’) and the reverse primer LSLPHP (5’ TTTGTTAACGGCGTCCGCGGCGGT 3’). The amplified product was digested with the restriction enzymes, BamH1 and Hpa1. The CAH1 small subunit was amplified using primers SUUP2 (5’ ACTAGTGCCGAGTCCGCGAAC 3’) and T3-2(5’ GCTATGACCATGATTACGCC 3’), and was digested with the restriction enzymes, Spe1 and Xba1. The following nucleotide sequences were obtained from Integrated DNA Technologies, Inc.

ASAAS2 :-

5’GTTAACCTGATGGTGCGCCGCTTCTACCACAAGCCCGAGTCCACCGCC

GCCCCTGTGGAGGAGCTGCACGCGCACAACCACCTGCTGCGCCGCCACC

ACCACCACGGCACTAGT 3’

ASAAS3:-

5’ACTAGTGCCGTGGTGGTGGTGGCGGCGCAGCAGGTGGTTGTGCGCGTG

CAGCTCCTCCACGGAGGCGGCGGTGGACTCGGGCTTGTGGTAGAAGCGG

CGCACCATCAGGTTAAC 3’

These single-stranded nucleotides were mixed together in equal proportion to form a duplex oligonucleotide which was later digested with the restriction enzymes, Hpa1 and Spe1 to obtain the modified spacer segment with reverse order of the amino acids. The pBSSK4 vector was digested with the restriction enzymes, Bamh1 and Xba1, and the four DNA fragments, containing, respectively, the large subunit plus ER targeting sequence, the spacer sequence, the small subunit sequence, and the pBSSK4 vector, were ligated to obtain the final Gr4Con1 construct.

Gr4Con2:

This construct has a modified spacer region with distinctly different amino acid sequence when compared to the positive control, Gr1Con1. During the process of generation of another construct, due to a PCR anomaly, the translation frame of the spacer region was shifted. Thirteen additional base pairs were added at the 5’ end of the spacer while eight base pairs were added at the 3’ end of the spacer effectively generating a spacer region with a distinctly different amino acid sequence. The nucleotide encoding this peptide had Sst1 restriction site at the 5’ end and a Spe1 restriction site at 3’ end. The large subunit along with ER targeting sequence was amplified using primers T7-2 (5’GACTCACTATAGGGCGAAT 3’) and LS5Sst1 (5’AAAAGAGCTCGGCGTCCGCGGC3’) and the resultant PCR product was digested with

BamH1 and Sst1 restriction enzymes. Gr4Con1 was digested with BamH1 and Spe1 restriction enzymes to generate a digested bluescript vector with attached *CAH1* small subunit sequence. The digested large subunit sequence, along with the ER targeting sequence and the modified spacer sequence were ligated with the digested pBSSK4 vector described above to generate the final construct Gr4Con2.

Gr4Con3:

A DNA fragment containing the ER targeting sequence, the large subunit of *CAH1* and the *CAH1* spacer was PCR amplified with a BamH1 restriction site at the 3' end from Gr1Con1 using the forward primer T7-2 (5' CGACTCACTATAGGGCGAAT 3') and the reverse primer SPLOWBAMH1 (5' TTGGATTCCAGCATCACGCGGCG 3'). This amplified sequence was digested with Nco1 and BamH1 to generate a DNA fragment encoding only the large subunit and the spacer. A second nucleotide sequence containing the spacer and small subunit was amplified using the forward primer SPUPBamH1 (5' TTGGATCCGGCCACCACCACCAC 3') and the reverse primer T3-2 (5' GCTATGACCATGATTACGCC 3') with a BamH1 site on the 5' end. This amplified sequence was digested with BamH1 and Xba1.To generate a pBSSK4 vector with ER targeting sequence, Gr1Con1 was digested with Nco1 and Xba1, and the three pieces were ligated to generate the final product, Gr4Con3, which included *CAH1* containing two spacer coding segments.

Group 5 constructs:

Construct Gr1Con1 in pBSSK4 was used as a template for the following PCR reactions. The forward primer T7-2 (5' CGACTCACTATAGGGCGAAT 3') and the reverse primer SLS1 (5' AAAGGATCCCGTGGAGTTGCACTC 3') for Gr5Con1 and SLS2 (5' AAAGGATCCGCACTCCTTCAGGCC 3') for Gr5Con2 were used to generate a truncated

portion of the large subunit and the ER targeting sequence with a BamH1 restriction site at the 3' end. This amplified sequence was digested with Nco1 and BamH1 restriction enzymes to obtain only the truncated portion of the large subunit with an Nco1 site at the 5' end and a BamH1 site at the 3' end. In a separate PCR reaction, the forward primer SPUPBamH1 (5' TTGGATCCGGCCACCACC ACCAC 3') and the reverse primer T3-2 (5' GCTATGACCATGATTACGCC 3') were used to obtain the nucleotide sequence that contained the spacer and the small subunit with a BamH1 site at the 5' end. This amplified sequence was digested with BamH1 and Xba1 restriction enzymes to yield a fragment containing the spacer and small subunit.

For Gr5Con3 and G5Con4 constructs, a fragment containing the ER targeting sequence, together with the large subunit and the spacer sequence of CAH1 was PCR amplified with a BamH1 restriction site at the 3' end from Gr1Con1 using the forward primer T7-2 (5' CGACTCACTATAGGGCGAAT 3') and the reverse primer SPLOWBAMH1(5' TTGGATTCCAGCATCACGCGGCG 3'). This amplified sequence was digested with Nco1 and BamH1 restriction enzymes to obtain only the large subunit and the spacer with Nco1 at 5' end and BamH1 at 3' end. In the second PCR reaction, the forward primer SSS1 (5' TTT GGA TCC AAC CCC GAT GCC TAC 3') for Gr5Con3 and 13CASS (5' TTT GGA TCC TGC AAG GCC GTT GCC 3') for Gr5Con4 and the reverse primer T3-2 (5' GCTATGACC ATGATTACGCC 3') were used to obtain the nucleotide sequence that contained truncated portion of the small subunit with a BamH1 site at the 5' end, which was digested with BamH1 and Xba1.

These fragments were ligated together with the vector containing the ER targeting sequence to obtain final constructs.

Group 6 constructs:

Construct Gr1Con1 in pBSSK4 was used as a template for the following PCR reactions. The forward primer T7-2 (5’ CGACTCACTATAGGGCGAAT 3’) and the reverse primer LSBamH1 (5’ TTGGATCCGGCGTCCGCGGT 3’) for Gr6Con1 and Gr6Con2, SLS2 (5' AAA GGA TCC GCA CTC CTT CAG GCC 3') for Gr6Con3 and Gr6Con4 respectively were used to amplify large subunit while the forward primer Sh6CA3 (5’ AAGGATCCGCCGAGTCCGCGAAC 3’) for Gr6Con1 and Gr6Con3, and the forward primer SSS1 (5' TTT GGA TCC AAC CCC GAT GCC TAC 3') for Gr6Con2 and Gr6Con4 respectively along with the reverse primer T3-2 (5’ GCTATGACCATGATTACG CC 3’) were used to amplify the small subunit.

The fragments with large subunit were digested with Nco1 and BamH1 while the fragments with small subunit were digested with BahH1 and Xba1. These digested fragments were ligated with the predigsted BSSK4 vector with BamH1 and Xba1 containing the ER targeting sequence.

Insertion of the constructs into the binary vector, pCB302-3

The binary vector, pCB302-3(Xiang et al., 1999), which contains a Kanamycin resistance cassette for selection of transformed *E.coli* and a Bar gene for selection of transgenic plants, was used in this research. This binary vector has a multi-cloning site flanked by a 35S promoter for constitutive expression of the construct in the plants and a Nos terminator.

After the successful transformation into *E. coli* strain Dh5α, each of the above described vectors was isolated and purified using commercially available QIAquick vector purification kit (Qiagen), and the coding sequence was confirmed by sequencing the entire construct using the

forward T7-2 (5' CGACTCACTATAGGGCGAAT 3') and reverse (5' GCTATGACCATGATTACGCC 3') primers that lie outside of the coding sequence. All the constructs in group 1 and group 2 and Gr3Con3, Gr3Con4, Gr3Con5, Gr3Con6, Gr4Con1 and Gr4Con2 constructs were excised from the purified pBSSK4 vector using BamH1 and Xba1 restriction enzymes. The resultant piece was ligated with the BamH1-Xba1 digested pCB302-3 vector.

Rest of the constructs contained two BamH1 restrictions sites in their coding sequence. Thus the following strategy was applied to insert a unique restriction site, Spe1, at their 5' end in the pBSSK4 vector. Two small "linker" nucleotides, PL1 (5' AGCTTACTGTCACTAGTGTAGCTC 3') and PL2 (5' TCGAGAGCTACACTAGTGACAGTA 3'), were synthesized in the ISU DNA synthesis facility and were mixed in equal proportions to obtain a double stranded oligonucleotide. The resultant double stranded oligonucleotide contained a digested Xho1 site on the 5' end and a HindIII site on the 3' end with an intact Spe1 restriction site in the middle. Each pBSSK4 vector containing the above constructs was digested with the restriction enzymes, Xho1 and HindIII, and ligated with the double stranded oligonucleotide. All of the resultant pBSSK4 vectors had a Spe1 restriction site at the 5' end and Xba1 at 3' end of the coding sequence. Each vector was then cut with Spe1 and Xba1 and the resultant piece was ligated into the Spe1-Xba1 digested pCB302-3 vector.

The successful ligation of the construct with the binary vector was confirmed by amplifying part of *CAH1* with the forward primer P7 (5' GAATTCGCCATGGCTTGCAT 3') and reverse primer P11 (5' GAGCTCTAGACTTTAGTGAT 3') using a standard colony PCR

method, and the amplified product was sequenced in ISU sequencing facility using the primer P3 (5’ ACTCCACCTCGGAGCACCTG 3’).
